# “Stromal cells in prostate cancer pathobiology: friends or foes?”

**DOI:** 10.1038/s41416-022-02085-x

**Published:** 2022-12-08

**Authors:** Filippo Pederzoli, Massimiliano Raffo, Hubert Pakula, Francesco Ravera, Pier Vitale Nuzzo, Massimo Loda

**Affiliations:** 1 Department of Pathology and Laboratory Medicine, New York Presbyterian Hospital, Weill Cornell Medicine, New York, NY USA; 2 Vita-Salute San Raffaele University, Milan, Italy; 3 Department of Internal Medicine, Università Degli Studi di Genova, Genova, Italy; 4 Meyer Cancer Center, Weill Cornell Medicine, New York, NY USA

**Keywords:** Prostate cancer, Cancer microenvironment

## Abstract

The genomic, epigenetic and metabolic determinants of prostate cancer pathobiology have been extensively studied in epithelial cancer cells. However, malignant cells constantly interact with the surrounding environment—the so-called tumour microenvironment (TME)—which may influence tumour cells to proliferate and invade or to starve and die. In that regard, stromal cells—including fibroblasts, smooth muscle cells and vasculature-associated cells—constitute an essential fraction of the prostate cancer TME. However, they have been largely overlooked compared to other cell types (i.e. immune cells). Indeed, their importance in prostate physiology starts at organogenesis, as the soon-to-be prostate stroma determines embryonal epithelial cells to commit toward prostatic differentiation. Later in life, the appearance of a reactive stroma is linked to the malignant transformation of epithelial cells and cancer progression. In this Review, we discuss the main mesenchymal cell populations of the prostate stroma, highlighting their dynamic role in the transition of the healthy prostate epithelium to cancer. A thorough understanding of those populations, their phenotypes and their transcriptional programs may improve our understanding of prostate cancer pathobiology and may help to exploit prostate stroma as a biomarker of patient stratification and as a therapeutic target.

## Introduction

Prostate adenocarcinoma (PCa) arises from the malignant transformation of the epithelial cells constituting the prostatic acini [1, 2]. Besides all the genomic, epigenomic and metabolic alterations occurring in neoplastic epithelial cells, a crucial role in PCa pathobiology is played by its ecosystem—known as the tumour microenvironment (TME). Prostate stroma is composed of a multitude of different cell populations—including fibroblasts, smooth muscle cells (SMCs), endothelium, immune cells and nerves—that actively contribute to its homoeostasis in physiologic and pathologic conditions, including PCa [2–4]. Indeed, stromal populations of prostate TME are known to be partners in crime with tumour cells, and they can represent a novel actionable target in the fight against cancer [5, 6].

It is not surprising that the stroma is central in PCa pathobiology, as it has played a pivotal role since its embryological development [7, 8]. Indeed, the prostate derives from the urogenital sinus (UGS), which is composed of an epithelial layer derived from the endoderm, surrounded by the embryonal prostatic mesenchyme derived from the mesoderm. Classical experiments using tissue recombination and grafting showed that the expression of the androgen receptor (AR) and paracrine factors (e.g. fibroblast growth factor (FGF) 7 and 10) in the mesenchymal UGS is necessary for the differentiation of embryonal epithelial cells into prostatic cells and branching ducts [9–11].

Through mechanisms similar to the ones in action during organogenesis, the activation of the mesenchymal stroma represents one of the first steps in prostate carcinogenesis [12, 13]. In this Review, we summarise the current knowledge about the stromal populations in the PCa microenvironment, describing the molecular pathways beyond stromal activation in PCa tumorigenesis and highlighting potential therapeutic strategies targeting the stroma.

## Fibroblasts

Fibroblasts represent the most studied and characterised cell population of prostate mesenchyme. In physiologic conditions, fibroblasts supervise the homoeostasis of extracellular matrix (ECM) and connective tissue, and they are also involved in tissue repair processes [14]. Embryologically, most prostate fibroblasts derive from the mesodermal mesenchyme, with a smaller proportion deriving from the neural crest [3]. Fibroblasts are generally recognised by their morphology and localisation within the tissue, besides the lack of epithelial, endothelial and hematopoietic markers. Indeed, some markers may be used for their identification, including fibroblast activation protein (FAP), platelet-derived growth factor receptor-α (PDGFRα), α-smooth muscle actin (αSMA) and vimentin [15, 16]. However, such markers are not exclusively expressed by bona fide fibroblasts. At the same time, some of them may preferentially designate specific fibroblast subpopulations (e.g. activated fibroblasts).

Fibroblasts are an active population within the stromal microenvironment, able to respond to various physiological and pathological stimuli by adapting their phenotype and behaviour. For instance, during wound healing, fibroblasts generally undergo a phenotypic differentiation towards contractile myofibroblasts and start releasing several cytokines and chemokines in the local microenvironment, including transforming growth factor-β (TGFβ) and vascular endothelial growth factor A (VEGFA) [17–19]. Similarly, noxious stimuli in a developing tumour push resting fibroblasts towards an activated status and are commonly referred to as cancer-associated fibroblasts (CAFs).

### Prostate fibroblasts in pre-malignant prostate lesions

Chronic inflammation and atrophic changes in prostatic tissue are present to a different extent in all aging men. While generally associated with benign prostatic hyperplasia (BPH) in the transitional zone, such alterations in the peripheral zone may represent the first steps toward carcinogenesis [20]. Indeed, several inflammatory conditions—including infections, dietary habits and hormonal changes—can modify the prostatic stroma into a pro-carcinogenic environment characterised by high concentrations of reactive oxygen species (ROS), inflammatory cells and pro-inflammatory soluble mediators [4, 20]. In this background, high-grade prostatic intraepithelial neoplasia (HG-PIN), considered the putative precursor lesions of PCa, may arise. Morphologically, HG-PIN is characterised by proliferating atypical epithelial cells with prominent nucleoli confined within the prostatic duct. Several alterations typical of chronic inflammation can be observed in the stroma surrounding HG-PINs. These include an increased number of vimentin + /α-SMA + myofibroblasts, signs of ECM remodelling and increased expression of procollagen I and tenascin C [12, 21, 22]. Fibroblasts’ transformation towards an activated, potentially noxious phenotype around HG-PIN lesions is sustained by soluble factors released by pre-malignant epithelial cells. Kryza et al. showed that PIN cells, like PCa cells, produce and release the serine protease kallikrein-related peptidase 4 (KLK4), which belongs to the same family of prostate-specific antigen (i.e. KLK3) [23]. KLK4 mediates paracrine effects within the TME through the activation of insulin-like growth factor (IGF) and TGF-β signalling pathways or the direct digestion of membrane-anchored proteins like protease-activated receptors (PARs) [23–26]. Indeed, KLK4 released by epithelial PIN cells can activate PAR1 expressed by prostate stromal cells, leading to increased production of pro-tumorigenic and pro-angiogenic factors, including FGF1, transgelin (TAGLN) and VEGF, which are all increased in the TME [27, 28]. Such evidence highlights the role of KLK4 in the crosstalk between prostatic epithelium and stroma in the early phase of PCa development.

### Prostate fibroblasts in prostate cancer

Compared to the normal fibromuscular stroma, PCa-associated reactive stroma is characterised by an increased proportion of fibroblasts/myofibroblasts, showing increased expression of vimentin, alpha-SMA, FAP, fibroblast-specific protein 1 (FSP-1) and desmin [3, 12, 29, 30], counterbalanced by the loss of differentiated SMCs and by ECM remodelling, e.g. increased production of tenascin and collagen type I [12, 29, 31]. Similar to normal fibroblasts, there are no universal markers for CAFs. For instance, FSP1 is also expressed by macrophages and some cancer cells, while desmin by pericytes [32–34]. CAFs’ immunophenotypic heterogeneity may be partially explained by their different precursors, including tissue-resident fibroblasts, mesenchymal stem cells, bone marrow-derived precursors and endothelial cells [35–38]. Indeed, integrating spatial, morphological and functional data is necessary to characterise the heterogeneity of CAFs in prostate TME, possibly shedding light on their influence on neoplastic epithelial cells.

CAFs can influence cancer cells’ fate through several mechanisms. The crosstalk between epithelial and stromal prostate cells is mediated by different paracrine soluble factors, of which TGF-β, “the molecular Jekyll and Hyde of cancer” [39], is probably the most studied and characterised [40]. Indeed, the expression of TGF-β is increased in both PIN and prostate cancer lesions, suggesting its key role in prostate TME since the first steps of carcinogenesis, and is consistent with the fibrotic and wound repair-like environment in PCa stroma [12, 41, 42]. Tuxhorn et al. showed in vitro that adding TGF-β1 to culture media promotes the phenotypic switch of HPS-TZ1A prostate stromal cells from normal fibroblasts to myofibroblasts. This phenotypic switch is blocked when TGF-β1 neutralising antibodies are added to the culture media [12]. Moreover, TGF-β1 promotes the production of vimentin from the HPS-TZ1A-transformed myofibroblasts. Other studies support the central role of TGF-β in modulating the ECM composition, for instance, by increasing the production of versican, an antiadhesive molecule that may facilitate the spreading of cancer cells [43]. Moreover, TGF-β constitutive overexpression in transgenic mice prostate results in developing an “aged” stroma characterised by fibroplasia and fibrogenous micronodules rich in tenascin and collagen [44]. These structures’ precise origin and function are unknown, but they are similar to collagenous micronodules described in human prostate cancer specimens [45].

In addition to TGF-β-driven ECM modifications, the reactive stroma is an active forge for the ECM’s structural, biochemical and biomechanical modifications around PCa foci [12, 46, 47]. CAFs actively shape prostate ECM mainly through three strictly interconnected mechanisms: overproduction of specific ECM molecules, release of ECM-remodelling matrix metalloproteinases (MMPs) and biomechanical and topographical modification of ECM fibres [48, 49]. Lipponen et al. described an increased deposition of hyaluronan, an anionic nonsulfated glycosaminoglycan, in the proximity of PCa foci, with higher levels of hyaluronan associated with aggressive features (e.g. high T stage, high Gleason score, perineural infiltration) [50]. Analysing the proteome of patient-matched CAFs and non-malignant fibroblasts from 4 radical prostatectomies, Nguyen et al. observed that the CAFs proteome was enriched in classes of proteins belonging to ECM and cell adhesion pathways [51]. In particular, network analyses in CAFs identified several collagen molecules (e.g. fibrillar COL1A1/2 and COL5A1 or non-fibrillar COL6A1 and COL7A1) and enzymes (e.g. LOXL2 and LOXL3) involved in ECM remodelling. Moreover, patient-derived CAFs cultured in vitro produced highly linearised ECM matrices compared to non-malignant fibroblasts, a conformational change in ECM morphology that generally favours tumour migration and dissemination [48]. Other biomechanical properties of CAFs have been associated with PCa. For instance, collagen fibre alignment was found higher in PCa than benign prostate tissues and directly associated with PCa aggressiveness and Gleason [52]. Among other non-collagenous ECM proteins, Erdogan et al. showed that CAFs promote PCa migration by remodelling the fibronectin network [46]. In vitro co-culture systems showed that fibronectin fibrils promote the formation of CAF/PCa cell units that migrate together. Moreover, CAFs promote the linearization of the fibronectin network by traction force on the ECM itself through a myosin II-PDGFRα-α5β1 integrin mechanism, which seems relevant in other stroma-rich tumours like pancreatic ductal adenocarcinoma.

Prostate TME is also characterised by an imbalance between matrix metalloproteinases and their inhibitors (i.e. tissue inhibitors of MMPs (TIMPs). Within the MMP family, MMP-2 and -9 and the inhibitory molecules TIMP-1 and -2 are well characterised in PCa [53–55]. Pro-MMP-2 is secreted in the prostate TME by stromal and cancer cells. It gets activated by enzymatic cleavage together with other local MMPs, like MMP-9, mediating ECM remodelling, cell migration and angiogenesis [55]. MMP-2 activity is regulated by several factors of epithelial and stromal origin. For instance, Wilson et al. showed that stromal cells, but not epithelial cells, produce pro-MMP-2 under in vitro standard conditions. Adding TGF-β to the culture medium leads to increased production of MMP-2 in both stromal and epithelial cells [54], suggesting its role in progression and invasion. Similarly, tumour-mesenchyme crosstalk regulates the activity of MMP-9, as shown in co-culture experiments using stromal (i.e. fibroblasts and SMCs) and PCa (primary and metastatic) cells [53]. Using this approach, Dong et al. showed a decreased expression of TIMP-1 and TIMP-2 in stromal cells, with increased production of pro-MMP-9 in PCa cells. Moreover, the regulation of MMP-9/TIMPs expression seems to be mediated by soluble factors rather than direct cell-to-cell interactions, with collagen I likely responsible for the increased production of pro-MMP-9 in PCa cells. Taken together, these data highlight the complex interplay between stroma and PCa cells in modifying the TME to favour or hinder tumour progression and invasion.

### Prostate cancer-associated fibroblasts in advanced and metastatic disease

In advanced stages, CAFs interactions with PCa cells mediate metastatic dissemination and response to therapy. Özdemir et al. used an intraosseous xenograft model transplanting osteoinductive human PCa cell lines (i.e. VCaP and C4-2B) in mice to investigate the different transcriptome of cancer and bone stromal cells [56]. Thus, they generated a transcriptional signature of the osteoblastic bone-metastasis associated stroma, whose most enriched pathways included angiogenesis and osteogenesis, ECM organisation and TGF-β receptor signalling. Notably, about 10% of stromal signature genes, including ASPN, POSTN and PDGFRB, was shared with PCa transcriptomes assessed in other studies, suggesting the phenotypic switch of the prostate TME towards a bone-like state in advanced PCa. Similarly, Tyekucheva et al. observed enrichment in bone-specific genes, including lumican, COL1A1 and biglycan, in microdissected stromal regions around high Gleason PCa foci [57]. Together with comparable evidence in other malignancies, these studies support the hypothesis that the stroma at the primary tumour site “trains and selects” for clonal tumour cells with a higher potential for site-specific metastatic tropism, thus making the primary TME an essential hotspot for therapeutic targeting and biomarker discovery.

Furthermore, CAFs can influence the response to several systemic therapies against PCa, including androgen deprivation therapy (ADT). AR, one of the most critical transcriptional programs in epithelial PCa cells, is a key transcription factor in stromal cells, likely responsible for canonical and cell-specific transcriptional activities. Indeed, Cioni et al. showed that, in CAFs, AR binds to chromatin regions that are not shared with PCa cells, thus influencing CAF-specific AR-driven transcriptional programs (e.g. regulation of pro-migratory cytokines release) [58]. Therefore, it is unsurprising that CAFs can play a role in the transition of PCa to a castration-resistant phenotype during ADT. Another way CAFs can influence the response to ADT is by promoting PCa cell progression towards neuroendocrine (NEPC) differentiation. Such transition has been described as a consequence of the expansion of a specific subpopulation of CAFs that promote NEPC transformation through Wnt-SFRP1 signalling [59], or an epigenetic-induced differentiation to NEPC through a glutamine-based metabolic rewiring [60]. Further studies are needed to dissect better the crosstalk between CAFs and PCa cells during systemic treatments.

### Shedding light on the heterogeneity of prostate stroma

Thanks to the advent of single-cell sequencing technologies, new efforts have been devoted to elucidating the heterogeneity of prostate stroma and the role of different stromal populations in PCa carcinogenesis. Oh-Joon Kwon et al. combined sequencing, flow cytometry and immunostaining to study the composition of the healthy adult mouse prostate stroma, identifying three major groups of cells (R1, R2 and R3) with different transcriptional programs and potential functions [16]. R1 subpopulation, primarily characterised by the surface markers (S1) Sca-1^+^/CD90^+^, showed low levels of vimentin expression and was characterised by an increased expression of genes involved in the Wnt pathway (e.g. *Wnt2*, *Wif1*, *Sfrp2*), ECM remodelling/morphogenesis (e.g. *Hoxd13*, *Bmp2*, *Bmp7*, *Mmp2*) and androgen biosynthesis (e.g. *Srd5a2*). Since R1 stromal cells are mainly located near epithelial cells, these cells are likely to play an essential role in the paracrine androgen signalling between stroma and epithelium. R2 subpopulation, mainly corresponding to Sca-1^+^/CD90^-/low^ (S2) cells, was characterised by high expression of *S100a4* and *Acta2*, together with other genes involved in complement activation, ECM remodelling and cytokine-/chemokine-mediated pathways. Altogether, these findings suggest that the R2 subpopulation may encompass myofibroblasts, involved in tissue repair and inflammation-mediated events. Lastly, the R3 subpopulation, which was not clearly distinguishable using flow cytometry panels, was associated with SMCs and was characterised by the expression of *Acta2*, *Tagln*, *Mfap2* and *Mfap4*. In addition to their scaffolding role within prostate parenchyma, R3 cells also expressed genes associated with prostate embryological development, and control of angiogenesis and neurogenesis, suggesting a much more complex role of these cells in the mesenchyme. Similar investigations in murine cancer models and clinical specimens are greatly awaited to further advance our understanding of stromal heterogeneity in prostate TME.

This heterogeneity in fibroblast phenotypes and functions may be partially explained by their spatial arrangement within the prostatic tissue. Berglund et al. performed a spatial transcriptomic analysis on different cores obtained from a radical prostatectomy specimen (Gleason score 3 + 4, pT3b). The study revealed significant spatial heterogeneity in both epithelial and stromal cells, confirming the existence of multiple CAFs subpopulations [61]. Normal stroma mostly expressed gene programs associated with the regulation of cytoskeleton and cell movement, complement activation and androgen signalling. In contrast, reactive stroma close to tumour foci was characterised by oxidative stress and integrin-linked kinase (ILK) signalling. Interestingly, some genes showed a gradient of expression from reactive stroma towards normal regions, further highlighting the importance of a proper, “pro-tumoral” environment before tumour cells can invade and proliferate.

### Translational relevance of reactive stroma in PCa therapeutic management

Histopathologically, PCa reactive stroma encompasses several TME alterations resulting from two opposite mechanisms: on one side, the remodelling of the host microenvironment mediated by cancer cells to support tumorigenesis; on the other, the antitumoral host response against cancer cells. Thus, it is not surprising that PCa reactive stroma has been assessed as a biomarker of PCa recurrence or progression in several studies (Table 1). In 2003, Ayala et al. reported that PCa reactive stroma was a predictive biomarker of biochemical-free survival [29]. Using Masson’s trichrome staining, they defined a 4-tier grading system (reactive stroma grading, RSG) based on the volume of the reactive stroma. Overall, the percentage of reactive stroma was a significant predictor of survival, showing the highest discriminative potential in tumours with Gleason scores 6 and 7. The histomorphologic characteristics of reactive stroma in hematoxylin-eosin-stained specimens and its predictive value have been confirmed in following studies conducted worldwide [62–70]. Given the unmet need for reliable factors for identifying aggressive and potentially lethal PCa cases, assessing stroma-based biomarkers and their combination with tumour-based ones is paramount to improving patient care. The reactive stroma around pre-neoplastic and neoplastic prostate lesions can be rigged by tumour cells to favour their proliferation and invasion. Therefore, it is not surprising that therapies targeting the reactive stroma have been tested in preclinical and clinical settings, especially in stroma-rich tumours like pancreatic cancer. Different strategies can be envisioned for this intent: (i) interfering with the creation of a pro-tumoral TME (e.g. stopping the recruitment of CAFs in tumour lesions, blocking ECM remodelling, inhibiting specific pathways involved in CAFs activation) [71, 72]; (ii) blocking the tumour-favourable interactions between CAFs and tumour cells (e.g. TGFβ, CXCR4 pathways) [73]; (iii) inhibiting the expression of immune suppressor factors (e.g. PD-L1) on mesenchymal stromal cells [74]; (iv) reverting the phenotype of CAFs to quiescent and/or tumour-restraining fibroblasts [75]; v) actively targeting CAFs through chimeric antigen receptor T (CAR T) cells or other cell therapy approaches [76]. Although many of these approaches are still at an early stage of clinical or preclinical development, developing novel agents targeting the TME may implement current therapeutic options to manage aggressive PCa, potentially improving patients’ clinical outcomes.

**Table 1:** Studies evaluating the reactive stroma as a biomarker in prostate cancer.

Author (year)	Main findings
Ayala et al., (2003; ref. 29.)	⁃ Development of the reactive stroma grading (RSG) system: ▫ RSG 0: tumours with 0–5% stroma ▫ RSG 1: tumours with 5–15% stroma ▫ RSG 2: tumours with 15–50% stroma ▫ RSG 3: tumours with >50% stroma ⁃ Evaluation of TMA cores by Masson’s trichrome stain from 545 patients ⁃ Volume of reactive stroma was a significant predictor of biochemical disease-free survival ⁃ Worst survival in patients with RSG 0 or RSG 3
Yanagisawa et al., (2007; ref. 68.)	⁃ RSG evaluated using H&E–stained sections of prostate biopsies from 224 patients ⁃ Patients with RSG 1 and 2 had better survival than those with RSG 0 and 3 ⁃ RSG was an independent predictor of recurrence ⁃ RSG is independent of Gleason 7 (either 4 + 3 or 3 + 4)
Ayala et al., (2011; ref. 65.)	⁃ Evaluation of the predictive value of the %RGS 3 in the entire tumour ⁃ 872 whole-mount, H&E–stained prostatectomy specimens ⁃ %RSG 3 was an independent predictor of biochemical recurrence ⁃ Higher %RSG 3 patients had a significantly decreased biochemical recurrence-free survival than those with lower %RSG 3
Billis et al., (2013; ref. 66.)	⁃ 266 H&E–stained needle prostatic biopsies ⁃ Increasing RSG was associated with higher clinical stage, preoperative PSA, Gleason score, and with more extensive tumours in radical prostatectomies ⁃ Only RSG 3 was associated with biochemical recurrence, but only on univariate analysis
Wu et al., (2014; ref. 67.)	⁃ 148 biopsies from patients with advanced PCa (cT3-cT4) before castration therapy, evaluated by Masson’s trichrome ⁃ RSG was inversely correlated with Gleason scores ⁃ Significant association between RSG and development of castration-resistant PCa in patients with initial Gleason score of 6–7
Saeter et al., (2015; ref. 64.)	⁃ Population-based study using H&E biopsies from 318 patients ⁃ RSG was associated with PCa-specific mortality in multivariate Cox regression analysis
McKenney et al., (2016; ref. 70.)	⁃ TMA cores from 1275 radical prostatectomies (Canary Retrospective Cohort) evaluated by H&E ⁃ Among the multiple architectural features assessed, cores were evaluated for presence/absence of RSG 3 ⁃ RSG 3 was associated with decreased post-surgery recurrence-free survival ⁃ RSG 3 was associated with worse survival when Gleason score 3 + 4 = 7 carcinomas alone were considered
Saeter et al., (2016; ref. 62.)	⁃ Population-based study using H&E biopsies from 318 patients ⁃ Perineural invasion (PNI) was associated with high RSG ⁃ The prognostic effect of PNI is dependent on an association with reactive stroma
Saeter et al., (2016; ref. 63.)	⁃ Population-based study using H&E biopsies from 283 patients ⁃ Patients with concomitant lymphovascular invasion (LVI) and high RSG were at high risk for PCa-specific death.
Ruder et al., (2022; ref. 69.)	⁃ Refinement of the original 4-tiers RSG to a binary system (qRS): ▫ qRS negative: tumours with <34% reactive stroma ▫ qRS positive: tumours with >34% reactive stroma ⁃ Algorithm-based quantification of reactive stroma ⁃ TMA cores from biopies and radical prostatectomies from different cohorts evaluated by H&E; > 1000 patients in total ⁃ qRS >34% was associated with worse outcomes (biochemical recurrence or PCa-specific death), after correcting for Gleason score and PSA

*H&E* hematoxylin-eosin staining, *PCa* prostate cancer, *PSA* prostate-specific antigen, *TMA* tissue microarray.

## Telocytes

Telocytes have been recently identified as a novel stromal cell type in several human organs [77–80]. Telocytes are characterised by an extensive network of thin extensions called telopodes, with specific phenotypic (CD34^+^, CD31^-^), transcriptional and morphological characteristics. In the prostatic gland, telocytes have been observed in the subepithelial regions, around the prostatic acini and within the interstitial stroma, in connection with local fibroblasts and SMCs [81–83]. Yet, their precise functions are largely unknown. Using the Mongolian gerbil (*Meriones unguiculatus*) model, Sanches et al. observed that ex vivo telocytes express the ER-β receptor and produce TGF-β1, respectively suggesting their potential sensitivity to oestrogens and their possible involvement in stromal cells differentiation around gerbil prostatic alveoli and ducts through paracrine effects [82]. The study also reported the formation of a structured telocyte network between birth and postnatal day 30, aimed at providing physical support for prostate compartmentalisation. In another report, Sanches et al. analysed the function of prostatic telocytes during aging, suggesting different roles for different subpopulations of telocytes [83]. On one hand, perialveolar telocytes maintain physical connections with SMCs throughout the aging process, potentially playing a role in the maintenance of stromal architecture. On the other, perivascular telocytes seem to participate in the onset of a reactive stromal environment, as they produce VEGF and express TNFα receptor. From such data, it appears that telocytes may play an active role in shaping the prostate mesenchyme, either by maintaining a physiological phenotype or by contributing to the age-related changes associated with BPH or PCa.

## Smooth muscles cells

In healthy conditions, SMCs are a constitutive component of the fibromuscular stroma that forms the prostatic parenchyma. Perturbations of the homoeostatic signalling network between SMCs and epithelial prostate cells have been reported in several prostatic diseases [84, 85]. Taboga et al. observed that bundles of SMCs were present around the epithelial structures in non-malignant prostate areas, while fewer and less organised SMCs appeared around prostate cancer foci, with an inverse correlation to Gleason score [86]. On an ultrastructural level, SMCs close to noninvasive cancer cells generally had a decreased organisation of the cytoskeleton, accumulation of peripheral vacuoles and loss of spatial intercellular connections, resulting in isolated cells surrounded by a thicker ECM. Concordantly to these observations, invasive cancer cells induced degenerative processes in SMCs, leading to cell atrophy, loss of basal membrane integrity, cytoskeletal instability and expansion of the perinuclear space. Moreover, along with PCa progression, well-differentiated SMCs are gradually substituted by activated fibroblasts and myofibroblasts [12, 87]. Using different tumour sublines derived from the original Dunning R3347 rat PCa cell line, Zechmann et al. found that hormone-sensitive tumours showed a slower growth rate when implanted orthotopically in the prostate compared to subcutaneous implantation [88]. Tumours growing in the two locations displayed different morphological characteristics: in the orthotopic tumours, tumour cells generally formed glandular structures in close interaction with surrounding SMCs; in the subcutaneous tumours, tumours mainly formed dedifferentiated aggregates with polypoid cancer cells. Similar findings were also confirmed by Wong et al. in a hormone-dependent rat model of prostate cancer, showing a spectrum of SMC dedifferentiation and structural derangements from normal prostate tissue to prostatic dysplasia, well-differentiated and invasive prostate cancer [89]. Such data suggest the potential role of SMCs in preserving prostate physiologic architecture during the early phases of carcinogenesis. Well-differentiated SMCs seem to contribute to restraining tumour growth and inhibit its progression. In this setting, the hedgehog (HH) pathway appears to play an essential role in determining SMCs behaviour in response to carcinogenesis. Yang et al. used three PCa murine models (PB-MYC, ERG/PTEN and TRAMP) to study the interplay between SMCs and HH pathway [87], observing a different pattern in each model. SMCs were reduced in the PB-MYC and ERG/PTEN models, counterbalanced by an accompanying increase in CAF-like cells, while expanded to some degree in TRAMP mice. HH signalling was intensely active in all SMCs close to PCa foci, with a low expression of Sonic Hedgehog and a high expression of Indian and Desert Hedgehog in epithelial PCa cells. Notably, elevated HH stromal signalling in PB-MYC resulted in increased SMCs around tumour foci, forming a physical barrier that prevented tumour progression and invasion. Further studies addressing the complexity of the tumour-stroma crosstalk are needed to understand better the HH pathway’s function in PCa pathobiology and its potential targeting in therapeutic strategies.

### AR signalling in SMCs

AR transcriptional activity in SMCs has been investigated with controversial observations. Using a transgenic mouse model (PTM-ARKO) with a specific AR knockout (KO) in SMCs, Welsh et al. found several alterations in the ventral lobes of KO mice [90]. KO adult mice had smaller prostates than controls, and histomorphologic abnormalities were present in the epithelial (i.e,. elongated epithelial folding, cell hypertrophy) and stromal (i.e., stromal hyperplasia, diffuse fibrosis) compartments. Upon stimulation by exogenous testosterone + estradiol-17β (T + E2), both controls and KO mice showed an increase in prostate volume, but the percentage increase in KO mice (213%) was significantly higher than in controls (25%). Moreover, controls and KO mice showed stromal and epithelial hyperplasia and hypertrophy upon hormonal stimulation. On the other hand, the reduction of the ventral lobe upon castration was lower in KO than in control mice, in line with previous studies suggesting a preponderant role of stromal rather than epithelial AR in mediating the response to castration [7]. The findings from the study by Welsh et al., however, did not wholly recapitulate the observations made by Yu et al. in another transgenic model (SM-ARKO), where they mostly observed histomorphologic defects due to defective epithelial proliferation through the deregulation of the IGF-1 signalling pathway, rather than increased apoptosis or dedifferentiation [91]. Recently, Liu et al. designed an inducible, SMC-specific CreER murine model to delete AR after prostate development (Myh11- CreER^T2^; AR^flox/Y^; R26R-CAG-EYFP/ + ) [92]. By crossing this model with Hi-Myc mice (ARR2/probasin-Myc) and inducing AR deletion, they observed an increased epithelial luminal cell proliferation and tumour progression, suggesting a tumour-suppressing role of stromal AR. Similar results were also obtained by inducing prostate carcinogenesis by T + E2 administration. Additionally, single-cell RNA sequencing performed on both models showed that AR stromal deletion favours the activity of an aggressive subpopulation of secretory luminal cells characterised by high PI3K pathway activity. The role of AR in sustaining the physiologic phenotype of SMCs is also suggested by studies conducted after castration. Indeed, Antonioli et al. studied the behaviour of SMCs in the ventral lobe of castrated rats, observing a transition in shape upon castration, from elongated to folded/spinous appearance [93]. In the time window they assessed—up to 21 days from castration—SMCs did not undergo significant phenotypic transformations, as suggested by the unchanged expression of surface markers (smooth muscle myosin heavy chain and alpha-actin). Other authors found loss of prostatic muscle bundles and phenotypic changes after 100 days from castration [94].

Likely, the inconsistent observations about SMCs and associated transcriptional programs (e.g. AR) may be explained by the different models used, different study timelines and different timing of Cre activation (constitutional vs. early postnatally vs. adulthood). Nevertheless, these data may suggest an evolving role of SMCs during PCa carcinogenesis and response to therapy in humans, a topic that deserves further investigation.

## Vasculature-associated cell populations

The vasculature network within the prostate, mainly composed of endothelial cells (ECs), pericytes and vascular muscle cells, plays a fundamental role in healthy prostate homoeostasis and tumour evolution.

### Endothelial cells

In healthy conditions, a delicate balance between pro- and anti-angiogenic factors governs the physiological turnover of vessel maintenance and repair [95]. The reorganisation of the vascular network is a hallmark of cancer, and tumour-associated vessels are phenotypically different from normal ones, showing an inconsistent pericyte coverage, aberrant branching and enhanced leakiness [96]. Activation of the so-called “angiogenic switch” in the tumour microenvironment is mediated by the increased production and release of several pro-angiogenic factors that can strongly influence the phenotype of local ECs. Among them, the heparin-binding protein vascular endothelial growth factor A (VEGF-A) is considered one of the most critical playmakers [97]. While VEGF-A is generally found at low concentrations in healthy prostate, its level increases both in human and murine PCa [98, 99]. Moreover, greater VEGF-A expression in human PCa samples correlates with higher disease stage, recurrence risk and death [99, 100]. Other players modulating EC phenotype in PCa include: fibroblast growth factors (FGFs), which may exert a promitotic influence on ECs; matrix metalloproteinases (e.g. MMP-2, MMP-7 and MMP-9), which control EC adhesion or detachment from the surrounding stroma; cyclooxygenase enzymes and their products (i.e. prostaglandins and thromboxanes), which can promote VEGF-A production, EC mobilisation and sprouting; specific microRNAs (miRNAs) like miR-296, which is generally overexpressed by PCa cells and regulates VEGF and PDGF receptors in ECs; tumour exosomes-derived sphingomyelin, which can promote EC migration, invasion and tube formation [101–105]. Of note, EC behaviour is influenced by androgens in a sex-specific manner [106]. Androgens increase angiogenic events in vitro and in vivo in male but not female ECs, and these sex-specific pro-angiogenic effects are VEGF-dependent. Additionally, exposure of male ECs to dihydrotestosterone increases the mRNA expression and surface levels of VEGF receptor 2 (i.e. KDR), supporting a pro-angiogenic role for androgens through KDR-mediated VEGF signalling. Based on these data, therapeutic strategies to target angiogenesis have been tested in PCa as monotherapies and combined regimens with different chemotherapeutic agents [107]. The main targets of anti-angiogenesis therapies have been VEGF-A and its receptors, as their expression was associated with poorer prognosis [99, 100, 108, 109]. However, results from clinical trials were not particularly successful in hormone-sensitive and castration-resistant PCa, highlighting the need for further clinical and preclinical studies.

ECs may also regulate the response to radiation therapy. Indeed, high expression of caveolin-1 in ECs decreased the efficacy of radiotherapy in a xenograft model of PCa, likely through hyperactivation of resistance mechanisms to radiation-induced apoptosis and stabilisation of the tumour-associated microvasculature [110]. Notably, caveolin-1 overexpression has been associated with radio-resistance in other tumours, like pancreatic cancer [111], suggesting the potential utility of strategies based on caveolin-1 inhibition to enhance radiotherapy efficacy.

### Pericytes

Pericytes are mainly considered vascular wall-associated cells, whose principal functions include stabilising neo-vessels into mature vasculature, preventing vascular leakiness and avoiding excessive sprouting [32, 112]. Despite being largely ignored compared to other cell types in the TME, pericytes share several properties with the progenitor cells, and in pathological conditions they can differentiate into muscle cells, myofibroblasts, chondrocytes and osteoblasts [113–115]. The PDGF-BB-PDGFRβ signalling pathway is pivotal in physiologic and pathologic conditions of pericytes biology. PDGF-BB can be released by several cells, including ECs, which use it to recruit pericytes at the sites of neoangiogenesis, and tumour cells, which release it to promote the detachment of pericytes from the vasculature, thus making tumour neo-vessels more disorganised and prone to tumour cell intravasation and metastatic spreading [116, 117]. Moreover, tumour-released PDGF-BB seems to build a chemoattractant gradient that induces pericytes to move towards tumour cells. Once detached from ECs and close to tumour cells, pericytes appear to differentiate into CAFs through a PDGF-BB-promoted pericyte–fibroblast transition [118]. To this end, high PDGFB expression in human cancers has been described as a negative prognostic marker for survival, and it positively correlates with increasing tumour-associated stroma [116]. Future studies will further address the role of pericytes in cancer invasion and metastasis and their phenotypic switch within the tumour microenvironment.

## Conclusions and future directions

**Fig. 1:**
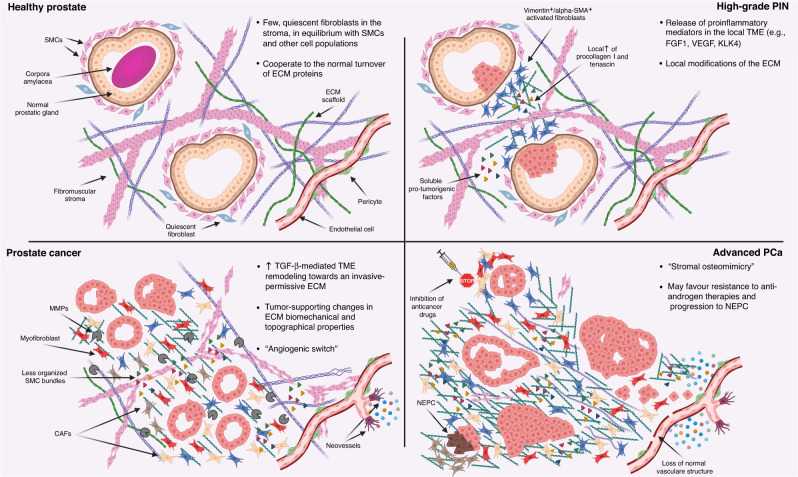
The stromal TME in prostate carcinogenesis. The complex interplay between cellular and acellular components of the stroma TME during prostate carcinogenesis. (alpha-SMA alpha-smooth muscle actin, CAF cancer-associated fibroblasts, ECM extracellular matrix, FGF1 fibroblast growth factor 1, KLK4 kallikrein-related peptidase 4, MMPs matrix metalloproteinases, NEPC neuroendocrine prostate cancer, PCa prostate cancer, PIN prostatic intraepithelial neoplasia, SMCs smooth muscle cells, TGF-β transforming growth factor β, TME tumour microenvironment, VEGF vascular endothelial growth factor).

It is now consensus that tumour pathobiology cannot be disentangled entirely without a thorough study of the tumour-associated microenvironment, of which stromal cells constitute a relevant proportion. In addition to tumour-specific genetic mutations, it is clear that the tumour-stroma interactions shape the processes of PCa tumorigenesis, progression and resistance to therapy (Fig. 1). Indeed, under selective pressure from anticancer therapies, cancer cells recruit the surrounding stroma to support their survival. There is no doubt that stroma-targeting strategies are needed to further increase our therapeutic armamentarium against PCa and improve patient outcomes, but some issues still need to be addressed. Firstly, therapies should be selective for cancer-associated stroma, thus requiring the identification of specific markers for targeted delivery, specific metabolic vulnerabilities for targeted interference and specific epigenetic programs for targeted modulation. Secondly, it is necessary to test different strategies combining tumour- and stroma-directed therapies, different timing of administration (sequential vs. combined) and different stages of disease (metastatic vs. locally advanced, progressing under standard-of-care therapies vs. de novo disease). Thirdly, stroma-based predictive and prognostic biomarkers should be tested, validated and introduced in clinical practice (e.g. the reactive stroma grading system for PCa reactive stroma) to refine patient stratification and potentially guide treatment decisions. Lastly, developing an ecologic and systemic perspective to study the TME in its cellular and acellular components would positively impact our understanding of the complex interplay between all its parts, further increasing our capabilities of targeting the TME for an overall improvement of patients’ management.

In conclusion, there is clear evidence that the stromal compartment of TME plays a fundamental role in cancer biology. Its specific interactions with the other TME components remain to be thoroughly elucidated. In the future, this field may uncover novel therapeutic approaches that target cancer-stroma system.

## Data Availability

Not applicable.

## References

[1] Pederzoli F, Bandini M, Marandino L, Ali SM, Madison R, Chung J (2020). Targetable gene fusions and aberrations in genitourinary oncology. Nat Rev Urol.

[2] Rebello RJ, Oing C, Knudsen KE, Loeb S, Johnson DC, Reiter RE (2021). Prostate cancer. Nat Rev Dis Prim.

[3] Sahai E, Astsaturov I, Cukierman E, DeNardo DG, Egeblad M, Evans RM (2020). A framework for advancing our understanding of cancer-associated fibroblasts. Nat Rev Cancer.

[4] Josson S, Matsuoka Y, Chung LWK, Zhau HE, Wang R (2010). Tumor-stroma co-evolution in prostate cancer progression and metastasis. Semin Cell Dev Biol.

[5] Olumi AF, Grossfeld GD, Hayward SW, Carroll PR, Tlsty TD, Cunha GR (1999). Carcinoma-associated fibroblasts direct tumor progression of initiated human prostatic epithelium. Cancer Res.

[6] Valkenburg KC, de Groot AE, Pienta KJ (2018). Targeting the tumour stroma to improve cancer therapy. Nat Rev Clin Oncol.

[7] Cunha GR, Ricke W, Thomson A, Marker PC, Risbridger G, Hayward SW (2004). Hormonal, cellular, and molecular regulation of normal and neoplastic prostatic development. J Steroid Biochem Mol Biol.

[8] Cunha GR, Vezina CM, Isaacson D, Ricke WA, Timms BG, Cao M (2018). Development of the human prostate. Differentiation..

[9] Donjacour AA, Thomson AA, Cunha GR (2003). FGF-10 plays an essential role in the growth of the fetal prostate. Dev Biol.

[10] Sugimura Y, Cunha GR, Bigsby RM (1986). Androgenic induction of DNA synthesis in prostatic glands induced in the urothelium of testicular feminized (Tfm/Y) mice. Prostate.

[11] Cunha GR, Lung B (1978). The possible influence of temporal factors in androgenic responsiveness of urogenital tissue recombinants from wild-type and androgen-insensitive (Tfm) Mice. J Exp Zool.

[12] Tuxhorn JA, Ayala GE, Smith MJ, Smith VC, Dang TD, Rowley DR (2002). Reactive stroma in human prostate cancer: induction of myofibroblast phenotype and extracellular matrix remodeling. Clin Cancer Res.

[13] Elo TD, Valve EM, Seppänen JA, Vuorikoski HJ, Mäkelä SI, Poutanen M (2010). Stromal activation associated with development of prostate cancer in prostate-targeted fibroblast growth factor 8b transgenic mice. Neoplasia..

[14] Gabbiani G (2003). The myofibroblast in wound healing and fibrocontractive diseases. J Pathol.

[15] Chang HY, Chi J-T, Dudoit S, Bondre C, van de Rijn M, Botstein D (2002). Diversity, topographic differentiation, and positional memory in human fibroblasts. Proc Natl Acad Sci USA.

[16] Kwon O-J, Zhang Y, Li Y, Wei X, Zhang L, Chen R (2019). Functional heterogeneity of mouse prostate stromal cells revealed by single-cell RNA-seq. iScience.

[17] Buechler MB, Turley SJ (2018). A short field guide to fibroblast function in immunity. Semin Immunol.

[18] Fukumura D, Xavier R, Sugiura T, Chen Y, Park EC, Lu N (1998). Tumor induction of VEGF promoter activity in stromal cells. Cell..

[19] Rockey DC, Weymouth N, Shi Z (2013). Smooth muscle α actin (Acta2) and myofibroblast function during hepatic wound healing. PLoS ONE.

[20] De Marzo AM, Platz EA, Sutcliffe S, Xu J, Grönberg H, Drake CG (2007). Inflammation in prostate carcinogenesis. Nat Rev Cancer.

[21] Ibrahim SN, Lightner VA, Ventimiglia JB, Ibrahim GK, Walther PJ, Bigner DD (1993). Tenascin expression in prostatic hyperplasia, intraepithelial neoplasia, and carcinoma. Hum Pathol.

[22] Xue Y, Smedts F, Latijnhouwers MA, Ruijter ET, Aalders TW, de la Rosette JJ (1998). Tenascin-C expression in prostatic intraepithelial neoplasia (PIN): a marker of progression?. Anticancer Res.

[23] Kryza T, Silva LM, Bock N, Fuhrman-Luck RA, Stephens CR, Gao J (2017). Kallikrein-related peptidase 4 induces cancer-associated fibroblast features in prostate-derived stromal cells. Mol Oncol.

[24] Ramsay AJ, Reid JC, Adams MN, Samaratunga H, Dong Y, Clements JA (2008). Prostatic trypsin-like kallikrein-related peptidases (KLKs) and other prostate-expressed tryptic proteinases as regulators of signalling via proteinase-activated receptors (PARs). Biol Chem.

[25] Mukai S, Yorita K, Yamasaki K, Nagai T, Kamibeppu T, Sugie S (2015). Expression of human kallikrein 1-related peptidase 4 (KLK4) and MET phosphorylation in prostate cancer tissue: immunohistochemical analysis. Hum Cell.

[26] Matsumura M, Bhatt AS, Andress D, Clegg N, Takayama TK, Craik CS (2005). Substrates of the prostate-specific serine protease prostase/KLK4 defined by positional-scanning peptide libraries. Prostate.

[27] Webber JP, Spary LK, Mason MD, Tabi Z, Brewis IA, Clayton A (2016). Prostate stromal cell proteomics analysis discriminates normal from tumour reactive stromal phenotypes. Oncotarget..

[28] Kosti I, Jain N, Aran D, Butte AJ, Sirota M (2016). Cross-tissue analysis of gene and protein expression in normal and cancer tissues. Sci Rep.

[29] Ayala G, Tuxhorn JA, Wheeler TM, Frolov A, Scardino PT, Ohori M (2003). Reactive stroma as a predictor of biochemical-free recurrence in prostate cancer. Clin Cancer Res.

[30] Öhlund D, Elyada E, Tuveson D (2014). Fibroblast heterogeneity in the cancer wound. J Exp Med.

[31] Barron DA, Rowley DR (2012). The reactive stroma microenvironment and prostate cancer progression. Endocr Relat Cancer.

[32] Armulik A, Genové G, Betsholtz C (2011). Pericytes: developmental, physiological, and pathological perspectives, problems, and promises. Dev Cell.

[33] Kikuchi N, Horiuchi A, Osada R, Imai T, Wang C, Chen X (2006). Nuclear expression of S100A4 is associated with aggressive behavior of epithelial ovarian carcinoma: an important autocrine/paracrine factor in tumor progression. Cancer Sci.

[34] Österreicher CH, Penz-Österreicher M, Grivennikov SI, Guma M, Koltsova EK, Datz C (2011). Fibroblast-specific protein 1 identifies an inflammatory subpopulation of macrophages in the liver. Proc Natl Acad Sci USA.

[35] Potenta S, Zeisberg E, Kalluri R (2008). The role of endothelial-to-mesenchymal transition in cancer progression. Br J Cancer.

[36] Quail DF, Joyce JA (2013). Microenvironmental regulation of tumor progression and metastasis. Nat Med.

[37] Kalluri R, Zeisberg M (2006). Fibroblasts in cancer. Nat Rev Cancer.

[38] Direkze NC, Hodivala-Dilke K, Jeffery R, Hunt T, Poulsom R, Oukrif D (2004). Bone marrow contribution to tumor-associated myofibroblasts and fibroblasts. Cancer Res.

[39] Bierie B, Moses HL (2006). Tumour microenvironment: TGFbeta: the molecular Jekyll and Hyde of cancer. Nat Rev Cancer.

[40] Ahmadi A, Najafi M, Farhood B, Mortezaee K (2019). Transforming growth factor-β signaling: tumorigenesis and targeting for cancer therapy. J Cell Physiol.

[41] Wikström P, Stattin P, Franck-Lissbrant I, Damber JE, Bergh A (1998). Transforming growth factor beta1 is associated with angiogenesis, metastasis, and poor clinical outcome in prostate cancer. Prostate.

[42] Steiner MS, Barrack ER (1992). Transforming growth factor-beta 1 overproduction in prostate cancer: effects on growth in vivo and in vitro. Mol Endocrinol.

[43] Sakko AJ, Ricciardelli C, Mayne K, Tilley WD, LeBaron RG, Horsfall DJ (2001). Versican accumulation in human prostatic fibroblast cultures is enhanced by prostate cancer cell-derived transforming growth factor β1. Cancer Res.

[44] Barron DA, Strand DW, Ressler SJ, Dang TD, Hayward SW, Yang F (2010). TGF-β1 induces an age-dependent inflammation of nerve ganglia and fibroplasia in the prostate gland stroma of a novel transgenic mouse. PLoS ONE.

[45] Epstein JI (2004). Diagnosis and reporting of limited adenocarcinoma of the prostate on needle biopsy. Mod Pathol.

[46] Erdogan B, Ao M, White LM, Means AL, Brewer BM, Yang L (2017). Cancer-associated fibroblasts promote directional cancer cell migration by aligning fibronectin. J Cell Biol.

[47] Levesque C, Nelson PS (2018). Cellular constituents of the prostate stroma: key contributors to prostate cancer progression and therapy resistance. Cold Spring Harb Perspect Med.

[48] Winkler J, Abisoye-Ogunniyan A, Metcalf KJ, Werb Z (2020). Concepts of extracellular matrix remodelling in tumour progression and metastasis. Nat Commun.

[49] Martinez-Vidal L, Murdica V, Venegoni C, Pederzoli F, Bandini M, Necchi A (2021). Causal contributors to tissue stiffness and clinical relevance in urology. Commun Biol.

[50] Lipponen P, Aaltomaa S, Tammi R, Tammi M, Agren U, Kosma VM (2001). High stromal hyaluronan level is associated with poor differentiation and metastasis in prostate cancer. Eur J Cancer.

[51] Nguyen EV, Pereira BA, Lawrence MG, Ma X, Rebello RJ, Chan H (2019). Proteomic profiling of human prostate cancer-associated fibroblasts (CAF) reveals LOXL2-dependent regulation of the tumor microenvironment. Mol Cell Proteom.

[52] Ling Y, Li C, Feng K, Palmer S, Appleton PL, Lang S (2017). Second harmonic generation (SHG) imaging of cancer heterogeneity in ultrasound guided biopsies of prostate in men suspected with prostate cancer. J Biophotonics.

[53] Dong Z, Nemeth JA, Cher ML, Palmer KC, Bright RC, Fridman R (2001). Differential regulation of matrix metalloproteinase-9, tissue inhibitor of metalloproteinase-1 (TIMP-1) and TIMP-2 expression in co-cultures of prostate cancer and stromal cells. Int J Cancer.

[54] Wilson MJ, Sellers RG, Wiehr C, Melamud O, Pei D, Peehl DM (2002). Expression of matrix metalloproteinase-2 and -9 and their inhibitors, tissue inhibitor of metalloproteinase-1 and -2, in primary cultures of human prostatic stromal and epithelial cells. J Cell Physiol.

[55] Wood M, Fudge K, Mohler JL, Frost AR, Garcia F, Wang M (1997). In situ hybridization studies of metalloproteinases 2 and 9 and TIMP-1 and TIMP-2 expression in human prostate cancer. Clin Exp Metastasis.

[56] Özdemir BC, Hensel J, Secondini C, Wetterwald A, Schwaninger R, Fleischmann A (2014). The molecular signature of the stroma response in prostate cancer-induced osteoblastic bone metastasis highlights expansion of hematopoietic and prostate epithelial stem cell niches. PLoS ONE.

[57] Tyekucheva S, Bowden M, Bango C, Giunchi F, Huang Y, Zhou C (2017). Stromal and epithelial transcriptional map of initiation progression and metastatic potential of human prostate cancer. Nat Commun.

[58] Cioni B, Nevedomskaya E, Melis MHM, van Burgsteden J, Stelloo S, Hodel E (2018). Loss of androgen receptor signaling in prostate cancer-associated fibroblasts (CAFs) promotes CCL2- and CXCL8-mediated cancer cell migration. Mol Oncol.

[59] Kato M, Placencio-Hickok VR, Madhav A, Haldar S, Tripathi M, Billet S (2019). Heterogeneous cancer-associated fibroblast population potentiates neuroendocrine differentiation and castrate resistance in a CD105-dependent manner. Oncogene..

[60] Mishra R, Haldar S, Placencio V, Madhav A, Rohena-Rivera K, Agarwal P (2018). Stromal epigenetic alterations drive metabolic and neuroendocrine prostate cancer reprogramming. J Clin Invest.

[61] Berglund E, Maaskola J, Schultz N, Friedrich S, Marklund M, Bergenstråhle J (2018). Spatial maps of prostate cancer transcriptomes reveal an unexplored landscape of heterogeneity. Nat Commun.

[62] Saeter T, Bogaard M, Vlatkovic L, Waaler G, Servoll E, Nesland JM (2016). The relationship between perineural invasion, tumor grade, reactive stroma and prostate cancer-specific mortality: a clinicopathologic study on a population-based cohort. Prostate..

[63] Saeter T, Vlatkovic L, Waaler G, Servoll E, Nesland JM, Axcrona K (2016). Combining lymphovascular invasion with reactive stromal grade predicts prostate cancer mortality. Prostate..

[64] Saeter T, Vlatkovic L, Waaler G, Servoll E, Nesland JM, Axcrona K (2015). The prognostic value of reactive stroma on prostate needle biopsy: a population-based study. Prostate..

[65] Ayala GE, Muezzinoglu B, Hammerich KH, Frolov A, Liu H, Scardino PT (2011). Determining prostate cancer-specific death through quantification of stromogenic carcinoma area in prostatectomy specimens. Am J Pathol.

[66] Billis A, Meirelles L, Freitas LL, Polidoro AS, Fernandes HA, Padilha MM (2013). Adenocarcinoma on needle prostatic biopsies: does reactive stroma predicts biochemical recurrence in patients following radical prostatectomy?. Int Braz J Urol.

[67] Wu JP, Huang WB, Zhou H, Xu LW, Zhao JH, Zhu JG (2014). Intensity of stromal changes is associated with tumor relapse in clinically advanced prostate cancer after castration therapy. Asian J Androl.

[68] Yanagisawa N, Li R, Rowley D, Liu H, Kadmon D, Miles BJ (2007). Stromogenic prostatic carcinoma pattern (carcinomas with reactive stromal grade 3) in needle biopsies predicts biochemical recurrence-free survival in patients after radical prostatectomy. Hum Pathol.

[69] Ruder S, Gao Y, Ding Y, Bu P, Miles B, De Marzo A (2022). Development and validation of a quantitative reactive stroma biomarker (qRS) for prostate cancer prognosis. Hum Pathol.

[70] McKenney JK, Wei W, Hawley S, Auman H, Newcomb LF, Boyer HD (2016). Histologic grading of prostatic adenocarcinoma can be further optimized: analysis of the relative prognostic strength of individual architectural patterns in 1275 patients from the canary retrospective cohort. Am J Surg Pathol.

[71] Lin HM, Lee BY, Castillo L, Spielman C, Grogan J, Yeung NK (2018). Effect of FAK inhibitor VS-6063 (defactinib) on docetaxel efficacy in prostate cancer. Prostate..

[72] Thompson CB, Shepard HM, O’Connor PM, Kadhim S, Jiang P, Osgood RJ (2010). Enzymatic depletion of tumor hyaluronan induces antitumor responses in preclinical animal models. Mol Cancer Ther.

[73] Domanska UM, Timmer-Bosscha H, Nagengast WB, Oude Munnink TH, Kruizinga RC, Ananias HJ (2012). CXCR4 inhibition with AMD3100 sensitizes prostate cancer to docetaxel chemotherapy. Neoplasia..

[74] Silzle T, Randolph GJ, Kreutz M, Kunz-Schughart LA (2004). The fibroblast: sentinel cell and local immune modulator in tumor tissue. Int J Cancer.

[75] Oberstein PE, Rahma OE, Beri N, Stoll-D’Astice AC, Duliege A-M, Nazeer S, et al. A phase 1b study evaluating IL-1β and PD-1 targeting with chemotherapy in metastatic pancreatic cancer (PanCAN-SR1). J Clin Oncol. 2022;40:557.

[76] Kakarla S, Chow KK, Mata M, Shaffer DR, Song XT, Wu MF (2013). Antitumor effects of chimeric receptor engineered human T cells directed to tumor stroma. Mol Ther.

[77] Zheng Y, Zhang M, Qian M, Wang L, Cismasiu VB, Bai C (2013). Genetic comparison of mouse lung telocytes with mesenchymal stem cells and fibroblasts. J Cell Mol Med.

[78] Zheng Y, Cretoiu D, Yan G, Cretoiu SM, Popescu LM, Wang X (2014). Comparative proteomic analysis of human lung telocytes with fibroblasts. J Cell Mol Med.

[79] Vannucchi M-G, Traini C, Manetti M, Ibba-Manneschi L, Faussone-Pellegrini M-S (2013). Telocytes express PDGFRα in the human gastrointestinal tract. J Cell Mol Med.

[80] Popescu LM, Faussone-Pellegrini M-S (2010). TELOCYTES—a case of serendipity: the winding way from Interstitial Cells of Cajal (ICC), via Interstitial Cajal-Like Cells (ICLC) to TELOCYTES. J Cell Mol Med.

[81] Corradi LS, Jesus MM, Fochi RA, Vilamaior PSL, Justulin- Jr LA, Góes RM (2013). Structural and ultrastructural evidence for telocytes in prostate stroma. J Cell Mol Med.

[82] Sanches BDA, Maldarine JS, Zani BC, Tamarindo GH, Biancardi MF, Santos FCA (2017). Telocytes play a key role in prostate tissue organisation during the gland morphogenesis. J Cell Mol Med.

[83] Sanches BDA, Tamarindo GH, dos Santos Maldarine J, da Silva ADT, dos Santos VA, Lima MLD (2020). Telocytes contribute to aging-related modifications in the prostate. Sci Rep.

[84] Bruengger A, Bartsch G, Hollinger BE, Holly B, Rohr HP (1983). Smooth muscle cell of the canine prostate in spontaneous benign hyperplasia, steroid induced hyperplasia and estrogen or tamoxifen treated dogs. J Urol.

[85] Cunha GR, Hayward SW, Dahiya R, Foster BA (1996). Smooth muscle-epithelial interactions in normal and neoplastic prostatic development. Acta Anat (Basel).

[86] Taboga SR, Scortegagna E, Siviero MP, Carvalho HF (2008). Anatomy of smooth muscle cells in nonmalignant and malignant human prostate tissue. Anat Rec (Hoboken).

[87] Yang Z, Peng YC, Gopalan A, Gao D, Chen Y, Joyner AL (2017). Stromal hedgehog signaling maintains smooth muscle and hampers micro-invasive prostate cancer. Dis Model Mech.

[88] Zechmann CM, Woenne EC, Brix G, Radzwill N, Ilg M, Bachert P (2007). Impact of stroma on the growth, microcirculation, and metabolism of experimental prostate tumors. Neoplasia..

[89] Wong YC, Tam NNC (2002). Dedifferentiation of stromal smooth muscle as a factor in prostate carcinogenesis. Differentiation..

[90] Welsh M, Moffat L, McNeilly A, Brownstein D, Saunders PTK, Sharpe RM (2011). Smooth muscle cell-specific knockout of androgen receptor: a new model for prostatic disease. Endocrinology..

[91] Yu S, Zhang C, Lin C-C, Niu Y, Lai K-P, Chang H-c (2011). Altered prostate epithelial development and IGF-1 signal in mice lacking the androgen receptor in stromal smooth muscle cells. Prostate.

[92] Liu Y, Wang J, Horton C, Yu C, Knudsen B, Stefanson J (2022). Stromal AR inhibits prostate tumor progression by restraining secretory luminal epithelial cells. Cell Rep.

[93] Antonioli E, Della-Colleta HHM, Carvalho HF (2004). Smooth muscle cell behavior in the ventral prostate of castrated rats. J Androl.

[94] Hayward SW, Baskin LS, Haughney PC, Foster BA, Cunha AR, Dahiya R (1996). Stromal development in the ventral prostate, anterior prostate and seminal vesicle of the rat. Acta Anat (Basel).

[95] Bergers G, Benjamin LE (2003). Tumorigenesis and the angiogenic switch. Nat Rev Cancer.

[96] Siemann DW (2011). The unique characteristics of tumor vasculature and preclinical evidence for its selective disruption by tumor-vascular disrupting agents. Cancer Treat Rev.

[97] Carmeliet P, Jain RK (2000). Angiogenesis in cancer and other diseases. Nature..

[98] Ferrer FA, Miller LJ, Andrawis RI, Kurtzman SH, Albertsen PC, Laudone VP (1997). Vascular endothelial growth factor (VEGF) expression in human prostate cancer: in situ and in vitro expression of VEGF by human prostate cancer cells. J Urol.

[99] Borre M, Nerstrøm B, Overgaard J (2000). Association between immunohistochemical expression of vascular endothelial growth factor (VEGF), VEGF-expressing neuroendocrine-differentiated tumor cells, and outcome in prostate cancer patients subjected to watchful waiting. Clin Cancer Res.

[100] Dvorak HF (2002). Vascular permeability factor/vascular endothelial growth factor: a critical cytokine in tumor angiogenesis and a potential target for diagnosis and therapy. J Clin Oncol.

[101] Kim CW, Lee HM, Lee TH, Kang C, Kleinman HK, Gho YS (2002). Extracellular membrane vesicles from tumor cells promote angiogenesis via sphingomyelin. Cancer Res.

[102] Gately S (2000). The contributions of cyclooxygenase-2 to tumor angiogenesis. Cancer Metastasis Rev.

[103] Doll JA, Reiher FK, Crawford SE, Pins MR, Campbell SC, Bouck NP (2001). Thrombospondin-1, vascular endothelial growth factor and fibroblast growth factor-2 are key functional regulators of angiogenesis in the prostate. Prostate.

[104] Würdinger T, Tannous BA, Saydam O, Skog J, Grau S, Soutschek J (2008). miR-296 regulates growth factor receptor overexpression in angiogenic endothelial cells. Cancer Cell.

[105] Kessenbrock K, Plaks V, Werb Z (2010). Matrix metalloproteinases: regulators of the tumor microenvironment. Cell..

[106] Sieveking DP, Lim P, Chow RWY, Dunn LL, Bao S, McGrath KCY (2010). A sex-specific role for androgens in angiogenesis. J Exp Med.

[107] Sarkar C, Goswami S, Basu S, Chakroborty D. Angiogenesis inhibition in prostate cancer: an update. Cancers (Basel). 2020;12:2382. 10.3390/cancers12092382 PMC7564110 32842503

[108] Kelly WK, Halabi S, Carducci M, George D, Mahoney JF, Stadler WM (2012). Randomized, double-blind, placebo-controlled phase III trial comparing docetaxel and prednisone with or without bevacizumab in men with metastatic castration-resistant prostate cancer: CALGB 90401. J Clin Oncol.

[109] Michaelson MD, Oudard S, Ou YC, Sengeløv L, Saad F, Houede N (2014). Randomized, placebo-controlled, phase III trial of sunitinib plus prednisone versus prednisone alone in progressive, metastatic, castration-resistant prostate cancer. J Clin Oncol.

[110] Klein D, Schmitz T, Verhelst V, Panic A, Schenck M, Reis H (2015). Endothelial Caveolin-1 regulates the radiation response of epithelial prostate tumors. Oncogenesis..

[111] Hehlgans S, Eke I, Storch K, Haase M, Baretton GB, Cordes N (2009). Caveolin-1 mediated radioresistance of 3D grown pancreatic cancer cells. Radiother Oncol.

[112] von Tell D, Armulik A, Betsholtz C (2006). Pericytes and vascular stability. Exp Cell Res.

[113] Humphreys BD, Lin SL, Kobayashi A, Hudson TE, Nowlin BT, Bonventre JV (2010). Fate tracing reveals the pericyte and not epithelial origin of myofibroblasts in kidney fibrosis. Am J Pathol.

[114] Mills SJ, Cowin AJ, Kaur P (2013). Pericytes, mesenchymal stem cells and the wound healing process. Cells..

[115] Dellavalle A, Maroli G, Covarello D, Azzoni E, Innocenzi A, Perani L (2011). Pericytes resident in postnatal skeletal muscle differentiate into muscle fibres and generate satellite cells. Nat Commun.

[116] Hosaka K, Yang Y, Seki T, Nakamura M, Andersson P, Rouhi P (2013). Tumour PDGF-BB expression levels determine dual effects of anti-PDGF drugs on vascular remodelling and metastasis. Nat Commun.

[117] Xian X, Håkansson J, Ståhlberg A, Lindblom P, Betsholtz C, Gerhardt H (2006). Pericytes limit tumor cell metastasis. J Clin Invest.

[118] Hosaka K, Yang Y, Seki T, Fischer C, Dubey O, Fredlund E (2016). Pericyte–fibroblast transition promotes tumor growth and metastasis. PNAS..

